# Vaginal lipidomics of women with vulvovaginal candidiasis and cytolytic vaginosis: A non-targeted LC-MS pilot study

**DOI:** 10.1371/journal.pone.0202401

**Published:** 2018-08-22

**Authors:** José Marcos Sanches, Paulo César Giraldo, Rose Amaral, Marcos Nogueira Eberlin, Lygia Azevedo Marques, Isabel Migliorini, Marcel Nakahira, Michel Jan Marinus Bieleveld, Michelle Garcia Discacciati

**Affiliations:** 1 Campinas State University, Department of Tocoginecology, Campinas, São Paulo, Brazil; 2 Campinas State University, Institute of Chemistry, Campinas, São Paulo, Brazil; 3 University of São Paulo, Polytechnic School, São Paulo, São Paulo, Brazil; 4 University of São Paulo, School of Pharmaceutical Sciences, São Paulo, São Paulo, Brazil; Universitat de Lleida-IRBLLEIDA, SPAIN

## Abstract

**Objective:**

To characterize the lipid profile in vaginal discharge of women with vulvovaginal candidiasis, cytolytic vaginosis, or no vaginal infection or dysbiosis.

**Design:**

Cross-sectional study.

**Setting:**

Genital Infections Ambulatory, Department of Tocogynecology, University of Campinas, Campinas, São Paulo–Brazil.

**Sample:**

Twenty-four women were included in this study: eight with vulvovaginal candidiasis, eight with cytolytic vaginosis and eight with no vaginal infections or dysbiosis (control group).

**Methods:**

The lipid profile in vaginal discharge of the different study groups was determined by liquid chromatography-mass spectrometry and further analyzed with MetaboAnalyst 3.0 platform.

**Main outcome measures:**

Vaginal lipids concentration and its correlation with vulvovaginal candidiasis and cytolytic vaginosis.

**Results:**

PCA, PLS-DA and hierarchical clustering analyses indicated 38 potential lipid biomarkers for the different groups, correlating with oxidative stress, inflammation, apoptosis and integrity of the vaginal epithelial tissue. Among these, greater concentrations were found for Glycochenodeoxycholic acid-7-sulfate, O-adipoylcarnitine, 1-eicosyl-2-heptadecanoyl-glycero-3-phosphoserine, undecanoic acid, formyl dodecanoate and lipoic acid in the vulvovaginal candidiasis group; N–(tetradecanoyl)-sphinganine, DL-PPMP, 1-oleoyl-cyclic phosphatidic, palmitic acid and 5-aminopentanoic acid in the cytolytic vaginosis group; and 1-nonadecanoyl-glycero-3-phosphate, eicosadienoic acid, 1-stearoyl-cyclic-phosphatidic acid, 1-(9Z,12Z-heptadecadienoyl)-glycero-3-phosphate, formyl 9Z-tetradecenoate and 7Z,10Z-hexadecadienoic acid in the control group.

**Conclusions:**

Lipids related to oxidative stress and apoptosis were found in higher concentrations in women with vulvovaginal candidiasis and cytolytic vaginosis, while lipids related to epithelial tissue integrity were more pronounced in the control group. Furthermore, in women with cytolytic vaginosis, we observed higher concentrations of lipids related to bacterial overgrowth.

## Introduction

The instability surrounding the vaginal ecosystem is a well-known fact in the gynecological practice, and such variations may derive from a variety of physiological or external factors. The vaginal microbiota is constituted by microorganisms that promote local environmental balance and its maintenance is established by complex interactions between the regular microbiota, the microbial metabolic products, the regular hormonal status and the host’s immune response [1].

A high number of women are affected by vulvovaginal diseases such as vulvovaginal candidiasis (VVC) and cytolytic vaginosis (CV). Both conditions present very similar symptomatology and, for this reason, they are commonly mistaken in the clinical practice, leading to misdiagnosis and inappropriate choice of treatment [2]. Nevertheless, subtle distinctions can be made between them. VVC produces a white and thick vaginal discharge, with vulvovaginal pruritus and eventually pain and vulvovaginal fissure. Its intense inflammatory process results from epithelium aggression caused by a fungus, usually *Candida albicans* [3]. On the other hand, CV contains no sort of infection; instead, it is a dysbiosis, consisting of an intense proliferation of lactobacillus that leads to cell lysis, histamine discharge and, ultimately, vaginal epithelial scaling. Rather than pruritus, CV patients often report vulvovaginal burning that is accentuated in the premenstrual period, thus mimicking vulvovaginal candidiasis. Moreover, CV is not accompanied by a cellular inflammatory process as observed in VVC [2,4,5].

The development of metabolome analysis through mass spectrometry allowed researchers to reach a correct interpretation of the quantitative and qualitative metabolic profile of one organism or biological system, considering the metabolites composition and dynamics with respect to genetic, physiological and environmental factors [6,7]. Lipidomics has emerged as a segment closely related to metabolomics and is dedicated to the global study of lipids, including their biochemical characteristics and networks formed within biological systems [8–11]. Nowadays, lipidomics studies are inclined to consider lipids as part of a broad integrated system of pathophysiological processes, instead of individual molecular structures with isolated functions [8].

The study of lipids composing the vaginal ecosystem is a new and promising perspective for better understanding gynecological conditions and their pathophysiological mechanisms. In this context, the objective of our study was to characterize the lipid profile in vaginal discharge samples of women with VVC, CV or no infection or dysbiosis, in order to enable improvement of diagnosis and treatment success.

## Methods

### Study population and clinical evaluation

A cross-sectional pilot study carried out at the Genital Infections Ambulatory of the Department of Tocogynecology of the University of Campinas included 24 sexually active, in contraceptive use, non-pregnant women aged 18 to 42 years. Experimental groups were divided and named as follows (n = 8): 1) VVC group: women with vulvovaginal candidiasis; 2) CV group: women with cytolytic vaginosis; 3) NL group: women without any type of vaginal infection or dysbiosis. The study was approved by the Ethics and Research Committee at the University of Campinas, CAAE n° 60648016.8.0000.5404, and written informed consent was obtained from all participants.

The participants were submitted to a specular examination for collection of vaginal samples by Dacron sterile swabs in order to perform lipid analyses and bacterioscopy by Gram stain. The characterization of the VVC group was based on the presence of yeast, pseudohyphae or blastospores in the vaginal discharge by microscopic examination by Gram stain, further confirmed by growth in Sabouraud culture media (Becton-Dickinson, Sparks, MD, USA). The criteria used for the diagnosis of CV included white and flocculated vaginal discharge accompanied by itching and/or burning at clinical evaluation, and Gram stain examination revealing presence of vaginal epithelial cell lysis, high number of *Lactobacillus* morphotypes, absence of leukocytes or microbial pathogens, and negative *Candida* sp culture.

Equivocal cases of endocervicitis, with presence of blood, bacterial vaginosis, trichomoniasis and/or mixed infections were ruled out. Bacterial vaginosis was diagnosed by the Amsel criteria [12] and Nugent score ≥ 7[13]. Infection by *Trichomonas vaginalis* was identified by the visualization of inflammatory cells in the vaginal smear by bacterioscopy (Gram stain) and predominance of coccoid and coccobacillary bacteria, as well as the visualization of the protozoan in fresh microscopy.

### Lipid analysis of the vaginal samples

Two samples of the vaginal content of each patient were collected using sterile swabs and immediately stored in dry 10-mL tubes at -80°C, until processing.

For extraction of lipids, the samples were randomized and each sample was resuspended in 1 mL of 1:2 CHCl_3_: MeOH solution (Sigma, Basel, Switzerland), followed by the addition of 0.33 mL of CHCl_3_ and 0.33 mL of deionized water. The extraction was made in 15-mL glass tubes. The solution was then stirred for 5 minutes, followed by centrifugation at 13,000 rpm for 5 minutes. The supernatant was discarded, and the bottom layer of the sample containing the lipid fraction was transferred to 1.5-mL glass tubes. All samples were dried using SpeedVac for 30 minutes at 30°C and kept frozen at -80°C until the date of analysis.

### Data acquisition

Lipid chromatographic separation was performed by ultra-high performance liquid chromatography (UHPLC) Agilent 1290 Infinity system (Agilent, Santa Clara, California, USA) and chromatographic elution was performed on Kinetex C18 column (4.6 mm x 50 mm x 2.6 μm) (Phenomenex, Torrance, CA, USA). For the positive ion mode, the aqueous mobile phase A solvent was 0.1% formic acid and phase B solvent was methanol; for the negative ion mode, phase A solvent was 5 mM Ammonium Acetate and phase B solvent was methanol. Before injection, the samples were randomized and inserted in the properly order. The mobile phase flow rate was 0.3 mL min^-1^ and the injection volume was 2 μL. The mobile phase gradient started at 5% of phase B changing linearly to 95% of phase B within 15 minutes and then returning to the initial composition, at which the gradient was kept constant for 5 minutes until the next run. This gradient profile was used for both positive and negative ion modes.

### Mass spectrometry

To obtain the mass spectra of samples in positive and negative ion modes, a hybrid mass spectrometer with QTOF 6550 mass analyzer (Agilent, Santa Clara, California, USA) was used. The instrumental parameters of the electrospray ionization source used in this study for both positive and negative ion modes were: VCap of 3,000 V; 100 V shredder voltage, 65 V skimmer voltage, 750 V OCT 1 RF Vpp, 290°C Gas Temperature, 350°C Sheath Gas Temperature, 12 L.min^-1^ Sheath Gas Flow. The mass spectra were acquired in centroid mode and the mass range used for acquisition was 50–1700 Da.

### Data processing

The raw data obtained was converted to the mzData format using the MassHunter Qualitative software (Agilent, Santa Clara, California, USA), eliminating isotopic interference. After conversion, the files were imported into the XCMS online software[14] for peak detection, alignment, retention time correction and other relevant pre-processing steps.

The data obtained from the online XCMS software was converted into an Excel table. Data normalization, scaling, hierarchical clustering into heatmaps and multivariate statistical analysis were performed in the MetaboAnalyst 3.0 platform [15]. Metabolites with a value of p < 0.05 (ANOVA) were considered representative for further investigation. Exploratory multivariate data analysis was performed using the Principal Component Analysis (PCA) unsupervised method and the Partial Least Squares Discriminant Analysis (PLS-DA), allowing selection of molecules with a VIP index higher than 1.0 as potential biomarkers. Putative lipid identification of the selected biomarkers was performed by measurement of their exact mass, retention time and elution profile, and further matching of such compounds in METLIN [14], Human Metabolome Database (HMDB) [16] and Lipid Maps databases [17].

## Results

### PCA and PLS-DA analyses

**Fig 1:**
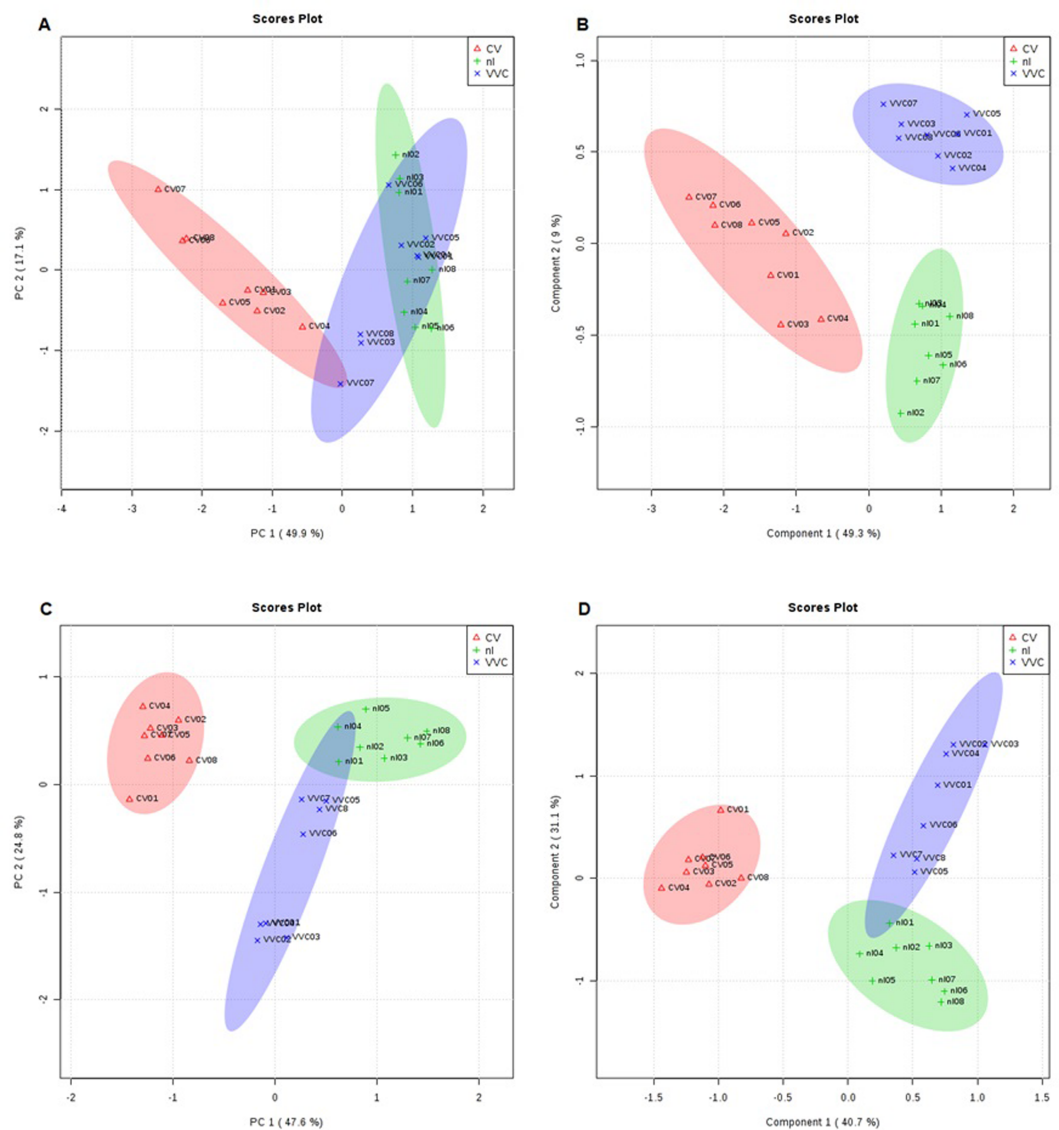
Score plots analysis of the lipid fraction of vaginal samples from CV (red), nl (green) and VVC (blue) groups. A: PCA analysis in the positive ion mode. C: PCA analysis in the negative ion mode. B: PLS-DA analysis in the positive ion mode. D: PLS-DA analysis in the negative ion mode.

Comparison between VVC, CV and nl groups with regard to the main lipid components was performed by PCA and PLS-DA analyses (Fig 1). Fig 1A and 1C refer to the PCA in positive and negative ion modes, respectively. Both graphs show that the CV ellipses are separated from the other groups, indicating that women with CV manifest a very distinct lipid composition. Larger overlaid area of VVC and nl ellipses in the positive ion mode characterizes a certain similarity between these groups. In the PLS-DA Parameter Score charts (Fig 1B and 1D), separation of the CV group from the VVC and nl groups was more pronounced when compared to the PCA, and this difference was probably due to the unsupervised nature of the latter method.

**Fig 2:**
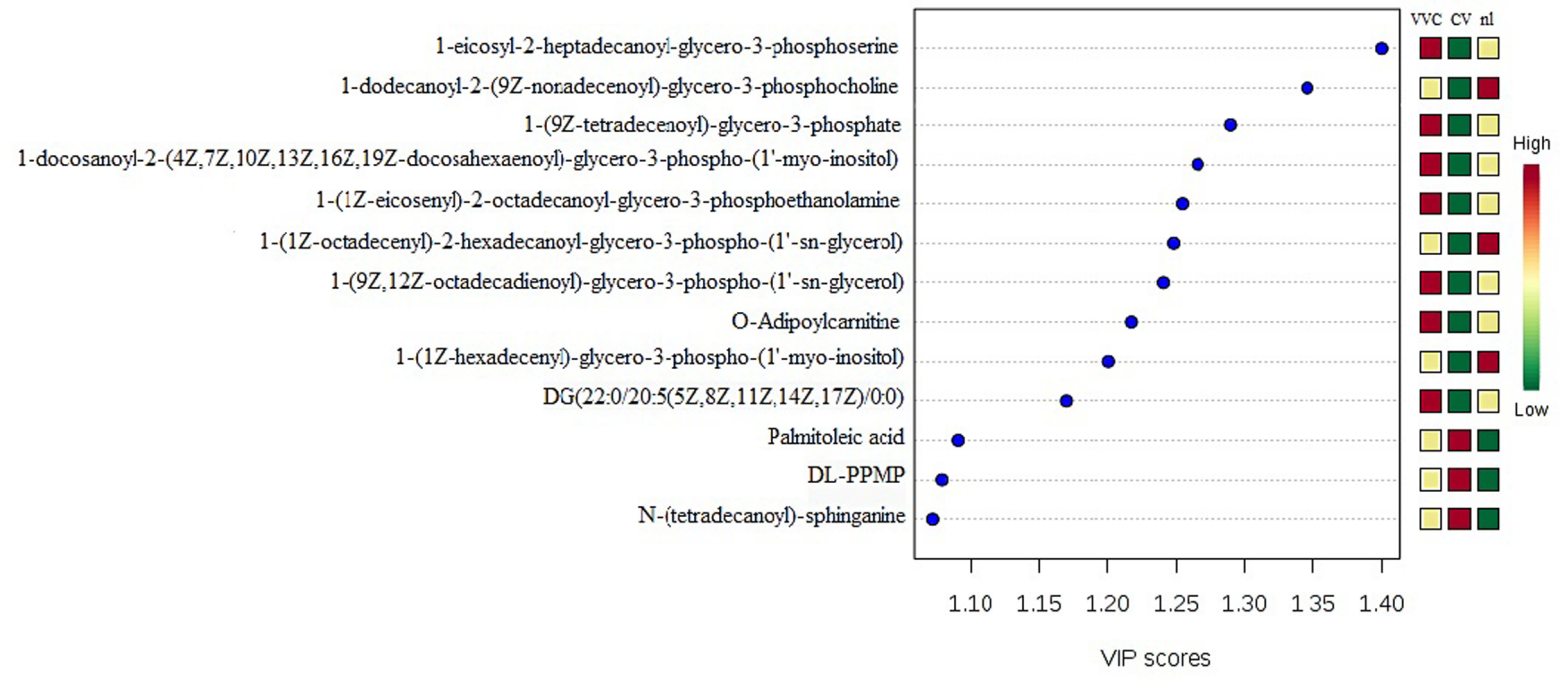
PLS-DA analysis in the positive ion mode revealing the 15 lipid molecules with the highest VIP scores and their respective concentrations in the vaginal samples from CV, nl and VVC groups.

**Fig 3:**
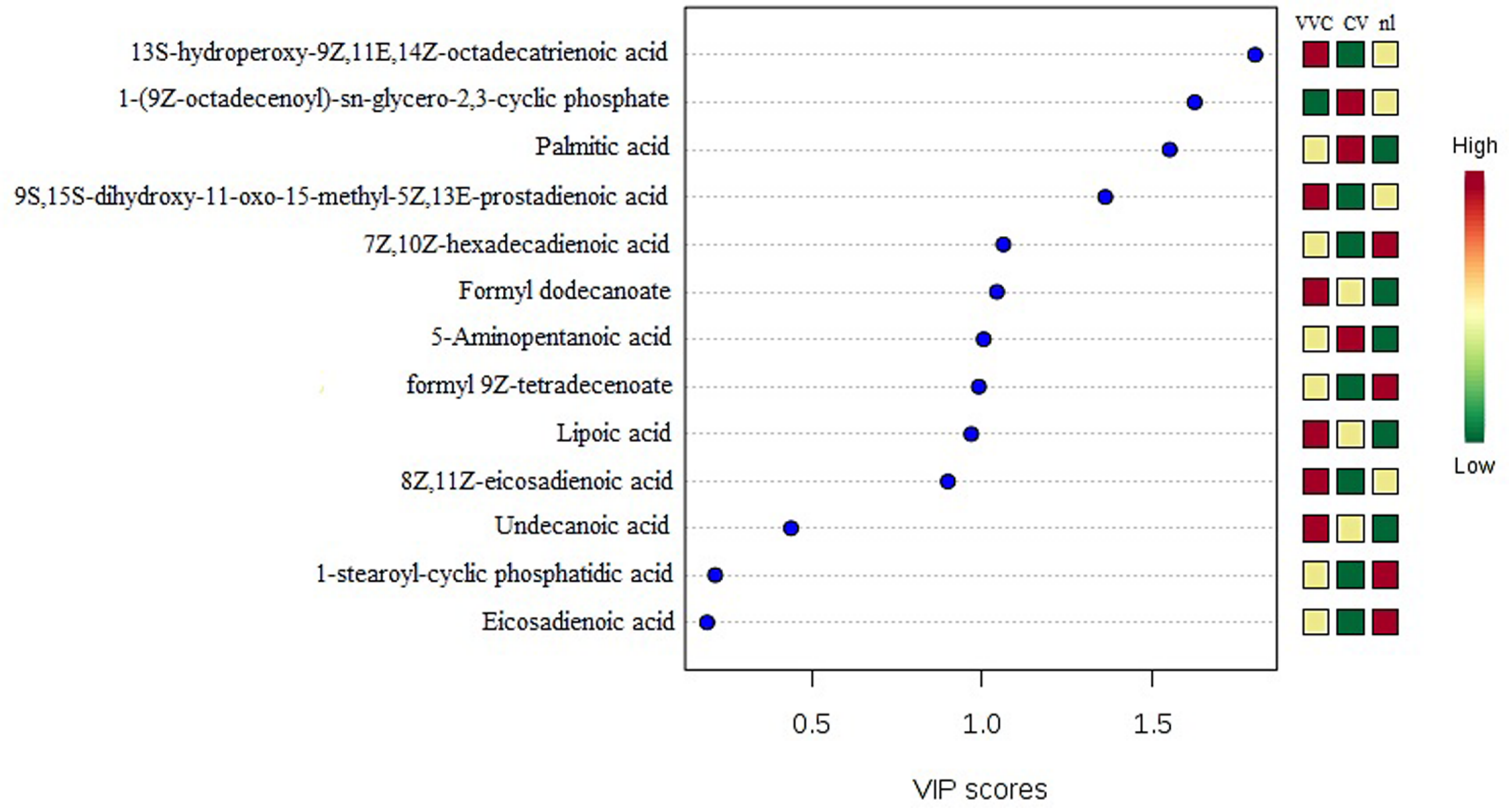
PLS-DA analysis in the negative ion mode revealing the 15 lipid molecules with the highest VIP scores and their respective concentrations in the vaginal samples from CV, nl and VVC groups.

The PLS-DA model also provides the VIP score, which is a measure of a variable's importance in the analysis. Figs 2 and 3 show the lipids with the highest VIP scores and their respective concentrations in the vaginal samples. As these molecules were the most contributory in the model, they were selected as potential biomarkers for the different experimental groups.

### Hierarchical clustering of lipids concentration by sample

**Fig 4:**
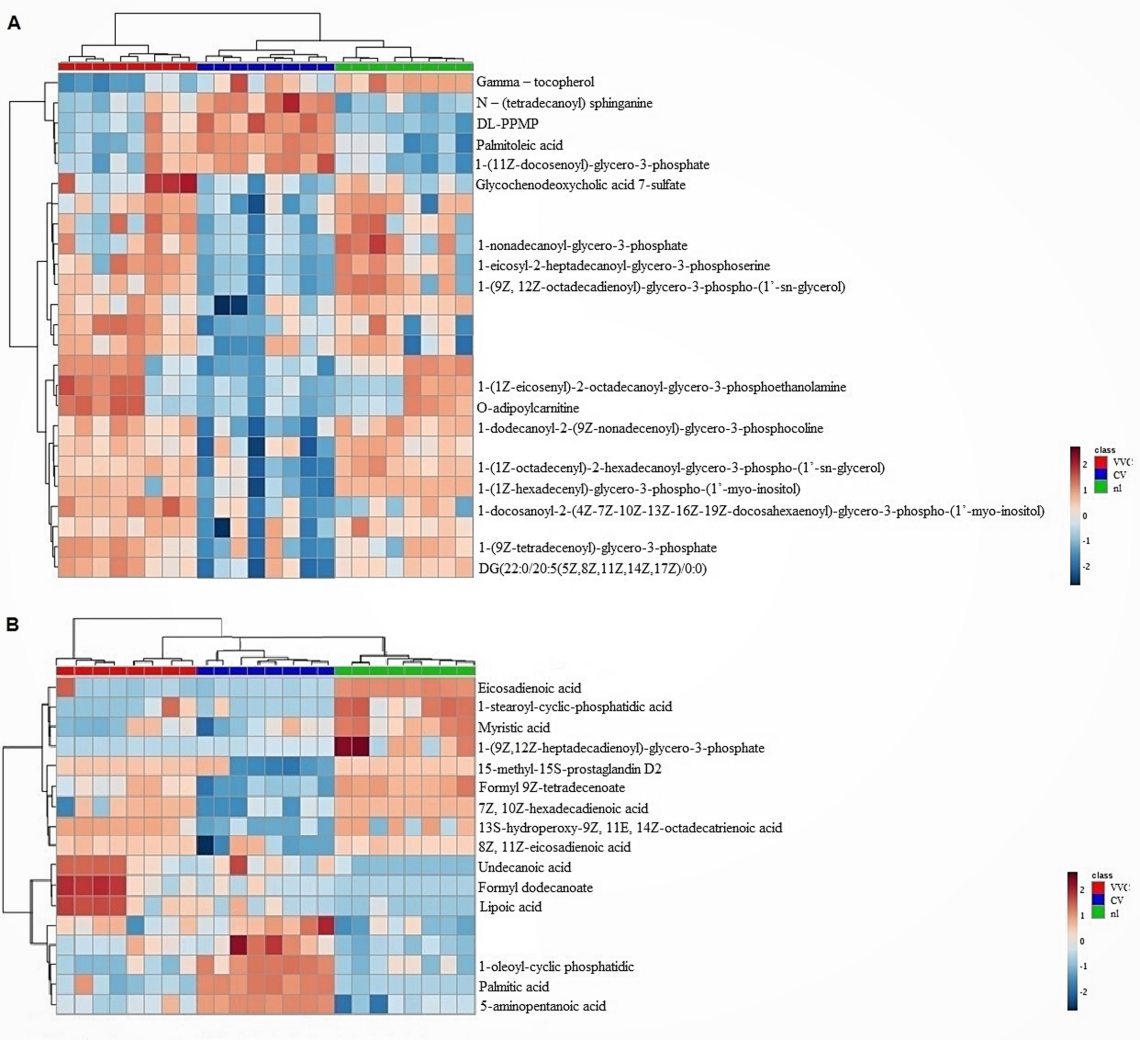
Hierarchical clustering of lipids with regard to their relative concentration in each vaginal sample from VVC (red dendrogram columns), CV (blue dendrogram columns) and nl (green dendrogram columns) groups (n = 8). High concentrations of lipids are shown in shades of red and low concentrations are shown in shades of blue. A: analysis performed in the positive ion mode. B: analysis performed in the negative ion mode.

Fig 4 shows the hierarchical clustering of lipids in the form of heatmaps and dendrograms, demonstrating the proportion of significantly altered lipid components, identified as potential lipid biomarkers of the studied groups. Results from this analysis confirmed some of the lipid proportions already evidenced by the former methods, as described below.

In VVC women, there was a higher concentration of Glycochenodeoxycholic acid 7-sulfate, 1-(1Z-eicosenyl)-2-octadecanoyl-glycero-3-phosphoethanolamine, O-adipoylcarnitine, 1-eicosyl-2-heptadecanoyl-glycero-3-phosphoserine in comparison with the control and CV groups. On the other hand, CV women showed higher concentrations of N–(tetradecanoyl) sphinganine, DL-PPMP and 1-(11Z-docosenoyl)-glycero-3-phosphate in relation to the other groups. The main potential lipid biomarkers of the control group (nl) were identified as 1-nonadecanoyl-glycero-3-phosphate and 1-(9Z, 12Z-octadecadienoyl)-glycero-3-phospho-(1’-sn-glycerol). Several lipids presented similar concentrations especially between the VVC and nl groups, thus corroborating the overlapping ellipses found in the PCA and PLS-DA analyses.

Moreover, the negative ionization mode showed the highest contrasts of lipid concentrations for VVC and CV groups. In this sense, the most expressive lipid biomarkers were undecanoic acid, formyl dodecanoate and lipoic acid for the VVC group, and 1-oleoyl-cyclic phosphatidic, palmitic acid and 5-aminopentanoic acid for the CV group. In parallel, eicosadienoic acid, 1-stearoyl-cyclic-phosphatidic acid, 1-(9Z,12Z-heptadecadienoyl)-glycero-3-phosphate, formyl 9Z-tetradecenoate and 7Z,10Z-hexadecadienoic acid were the main potential biomarkers for the control group.

Considering all methods of analysis, in both negative and positive ion modes, we found 38 potential biomarkers that allow distinction between the VVC, CV and control groups.

## Discussion

Our lipidomics study showed significant differences between women with VVC and CV regarding the lipid composition of vaginal discharge samples. Herein, we showed that lipids play an important role in the maintenance of the vaginal microenvironment homeostasis and that lipids profile can be markedly altered during pathophysiological processes. To our knowledge, this phenomenon had not yet been described in the current literature.

Moreover, several VVC potential lipid biomarkers were possibly related to inflammation. Among them, we found high concentrations of O-Adipoylcarnitine, belonging to the family of Acyl carnitines, which are intermediate oxidative metabolites synthesized by the mitochondria and peroxisomes, with the objective of transporting long-chain fatty acids for the process of β-oxidation [18,19]. Long-chain fatty acids can persistently modify biological processes, such as cellular stress [20], ionic variations [21] and inflammation [22]. Another lipid biomarker found in the VVC group, the 15-methyl-15S-prostaglandin D2 may act as a mediator of inflammation resulting from the vaginal mucosa response to the virulence factor of *Candida* sp.

Studies have shown that the oxidation of fatty acids typically occurs in tissues submitted to oxidative stress, including sites of inflammation [23,24]. Indeed, in the present investigation, a number of the selected biomarkers were found to be potentially related to oxidative stress. 13S-hydroperoxide-9Z,11E,14Z-octadecatrienoic acid, pointed in our study as the lipid biomarker with the highest VIP score in women with VVC (negative ion mode data), is typically produced by lipid peroxidation and can accumulate in mitochondrial membranes, enabling mitochondrial degradation and cellular damage [25–27]. Consistently, the glycochenodeoxycholic-acid-7-sulfate is the smallest of fatty acids metabolites whose excretion is increased when there are errors in mitochondrial metabolism, being suitable to diagnose mitochondrial beta-oxidation disorders [28, 29]. In our study, this compound was found in considerable proportions in the vaginal discharge of women with VVC and could be associated with mitochondrial deregulations in response to the oxidative stress caused by *Candida* sp.

A possible biological mechanism against oxidative stress and inflammatory process is the production of antioxidant metabolites or even by oral treatment, such as the lipoic acid [30, 31]. In its reduced form, dihydrolipoic acid, such compound has been described as a metabolite of fundamental importance to counterbalance reactive oxygen species [32–34]. In our study, lipoic acid was found as a potential biomarker of VVC, also possibly related to the vaginal mucosa response against *Candida* sp. oxidative stress.

Likewise, undecanoic acid was found at higher concentrations in the VVC group and seems to present antifungal and oxidative stress defense properties. This compound is an endogenous fatty acid commonly found in body fluids, and is involved in cell signaling, integrity and stability of biological membranes [35]. Studies have shown that the administration of undecanoic acid together with palmitic acid has resulted in antifungal activity [36] and inhibition of the morphogenesis of *Candida albicans*, preventing the development of blastospores into hyphae [37]. Thus, the presence of this lipid in women with VVC indicates a tissue response to *Candida* sp. infection.

Interestingly, palmitic acid was found at high concentrations only in the CV group. This compound is the most prominent fatty acid used by the human body [38]. Studies with rats have shown that palmitic acid increases endoplasmic reticulum stress, apoptosis in endothelial cells [39], increases endothelial nitric oxide synthase phosphorylation [40] and increases the formation of ROS and NADPH oxidase in skeletal muscles [41]. Therefore, CV lipid profile was also influenced by oxidative stress, probably caused by the exacerbated increase of lactobacilli producing lactic acid and other organic acids.

N-(tetradecanoyl) sphinganine and DL-threo-1-phenyl-2-palmitoylamino-3-morpholino-1-propanol (DL-PPMP) were also indicated as potential biomarkers for CV in our study. Both are precursors of apoptosis [42–44], possibly due to the homeostatic disturbance related to the low pH (3.5 to 4.5) that is characteristic of this vaginosis [5]. Additionally, the increase in the levels of palmitoleic acid and 1-oleoyl-cyclic phosphatidic acid in the CV group was consistent with the role of these acids on injured epithelial tissues, already described this relation in hepatic cells [45]. Our results showed the presence of these potential lipids biomarkers in CV group which can be inferred the possible relation of injured caused on the vaginal epithelium visualized on the Gram stain.

In the present report, lipid biomarkers such as the phosphatidylserines fatty acids phosphatidylinositol and phosphatidylglycerophosphate, involved in cell signaling such as apoptosis, signal transduction of the plasmatic membrane and cardiolipin precursors [35, 46], showed higher concentrations in the VVC group.

The 5-aminopentanoic acid can be produced endogenously or by the bacterial catabolism of lysine, and it is known to act on the growth of anaerobic bacteria [47, 48]. In contrast, the presence of this biomarker in high concentrations in the CV group, where there is predominance of lactobacilli, raises new questions about the function of this compound in situations where there is an overpopulation of aerobic bacteria.

Among the main lipids identified in the control group (nl), the eicosadienoic acid presented relevant concentrations in comparison to the other study groups. This compound is an omega-6 fatty acid whose synthesis is linked to several receptors that can be found in various tissues of the human body [35, 49, 50]. The eicosadienoic acid is an antagonist of the leukotriene B_4_ receptor [51], and both leukotrienes and prostanoids act in the body in autocrine and paracrine regulation, influencing many physiological and pathophysiological mechanisms within the cell [52]. This organic acid can be related to anti-inflammatory mechanisms exerting a protective control of inflammatory mediators [53]. In addition, the presence of glycerophospholipids as potential biomarkers in the control group supports the function normality of the vaginal epithelial tissue, as these lipids are commonly found in the human organism [35, 54].

Furthermore, tocopherols are lipids associated with the cell membranes, acting against the most reactive forms of free radicals, besides preventing lipid peroxidation and bacterial translocation, hence reducing tissue injuries [55–58]. Our study demonstrated a higher concentration of gamma-tocopherol in the control group, which is a derivative of vitamin E. Notably, this lipid is important for the vaginal epithelial tissue homeostasis as it acts as a protector against disorders in the local microenvironment, such as the oxidative stress caused by infections and dysbioses. Another antioxidant lipid biomarker found in the control group was myristic acid, which has been described as improving the bioavailability of polar antioxidant molecules in the organism [59], besides exerting antimicrobial activities [60].

## Conclusion

From the lipidomic point of view, we are facing three distinguishable vaginal profiles. In women with VVC, we found a higher concentration of lipids related to inflammation and oxidative stress, while in CV women, we observed a higher concentration of lipids related to cellular apoptosis, oxidative stress and bacterial overgrowth. In control women, vaginal lipidome was characterized by the presence of lipids involved in the maintenance of epithelial integrity epithelium and anti-inflammatory and antioxidant functions.

For clinical practice, this work provides some answers and new insights about the lipid metabolism involved in the pathophysiological processes of VVC and CV. Studies with larger population and targeting specifics lipids biomarkers can provide subsidies for better definition of conduct and treatment for women affected by these so frequent gynecological disorders.

## Details of ethics approval

The study was approved by the Ethics and Research Committee at the University of Campinas on 16 November 2012 (ref. no. 155.315), and written informed consent was obtained from all participants.

## Supporting information

S1 Database (XLSX)
